# Clozapine N-Oxide Administration Produces Behavioral Effects in Long–Evans Rats: Implications for Designing DREADD Experiments

**DOI:** 10.1523/ENEURO.0219-16.2016

**Published:** 2016-11-01

**Authors:** Duncan A. A. MacLaren, Richard W. Browne, Jessica K. Shaw, Sandhya Krishnan Radhakrishnan, Prachi Khare, Rodrigo A. España, Stewart D. Clark

**Affiliations:** 1 Department of Pharmacology and Toxicology, School of Medicine and Biomedical Sciences, University at Buffalo, SUNY, Buffalo, New York 14214; 2 Department of Biotechnical and Clinical Laboratory Sciences, University at Buffalo, SUNY, Buffalo, New York 14214; 3 Department of Neurobiology and Anatomy, Drexel University College of Medicine, Philadelphia, Pennsylvania 19129

**Keywords:** behavior, CNO, DREADDs, voltammetry

## Abstract

Clozapine N-oxide (CNO) is a ligand for a powerful chemogenetic system that can selectively inhibit or activate neurons; the so-called Designer Receptors Exclusively Activated by Designer Drugs (DREADD) system. This system consists of synthetic G-protein-coupled receptors, which are not believed to be activated by any endogenous ligand, but are activated by the otherwise inert CNO. However, it has previously been shown that the administration of CNO in humans and rats leads to detectable levels of the bioactive compounds clozapine and *N*-desmethylclozapine (*N*-Des). As a follow-up, experiments were conducted to investigate the effects of CNO in male Long–Evans rats. It was found that 1 mg/kg CNO reduced the acoustic startle reflex but had no effect on prepulse inhibition (PPI; a measure of sensorimotor gating). CNO (2 and 5 mg/kg) had no effect on the disruption to PPI induced by the NMDA antagonist phencyclidine or the muscarinic antagonist scopolamine. In locomotor studies, CNO alone (at 1, 2, and 5 mg/kg) had no effect on spontaneous locomotion, but 5 mg/kg CNO pretreatment significantly attenuated d-amphetamine-induced hyperlocomotion. In line with the behavioral results, fast-scan cyclic voltammetry found that 5 mg/kg CNO significantly attenuated the d-amphetamine-induced increase in evoked dopamine. However, the effects seen after CNO administration cannot be definitively ascribed to CNO because biologically relevant levels of clozapine and *N*-Des were found in plasma after CNO injection. Our results show that CNO has multiple dose-dependent effects *in vivo* and is converted to clozapine and *N*-Des emphasizing the need for a CNO-only DREADD-free control group when designing DREADD-based experiments.

## Significance Statement

Recently, interest in clozapine N-oxide (CNO) has increased due to its exploitation as a ligand for the engineered G-protein-coupled receptors (GPCRs) in the chemogenetic ‘Designer Receptors Exclusively Activated by Designer Drugs’ (DREADD) system. Our results highlight that in the experimental design there is a necessity for the inclusion of a group of animals which do not express DREADDs, but are given the same dose of CNO as the DREADD expressing animals. Currently, only a small minority of studies using DREADDs employ this control. There needs to be careful consideration of the CNO dose being administered and of the possible biological effects of CNO.

## Introduction

Clozapine N-oxide (CNO) is a major metabolite of the anti-psychotic drug clozapine. While clozapine is known to bind to many receptors (Coward, 1992; Schotte et al., 1993; Peters, 2012), the few studies that investigated the pharmacological actions of CNO failed to find any effects. This has led to the consensus that CNO is largely an inactive metabolite (Alves-Rodrigues et al., 1996; Salmi and Ahlenius, 1996; Wong et al., 1996). However, administration of CNO leads to detectable levels of clozapine in the plasma of humans and guinea pigs (Jann et al., 1994; Chang et al., 1998). The retroconversion of CNO to clozapine in rats is somewhat controversial as Jann et al. (1994) were unable to detect clozapine in the plasma of Wister rats after administration of CNO (1 mg/kg, i.p.), but Lin et al., 1996 were able to detect clozapine, *N*-desmethylclozapine (*N*-Des) and other minor clozapine metabolites in the urine of Lewis rats (20 mg/kg, oral; Lin et al., 1996). It is unclear whether the difference is due to differing strain, sample preparation, dosage, detection sensitivity, or due to one group assessing plasma and the other urine. It has been noted that species differences in the endogenous production of ascorbate, which perturbs CNO retroconversion *in vitro* (Pirmohamed et al., 1995) could help to explain the differences in CNO retroconversion. Nevertheless, there is evidence for reversible metabolism whereby clozapine is metabolized into *N*-Des and CNO, with a portion of the CNO being reduced back to the parent compound which presumably is then metabolized back into CNO in an ever diminishing cycle leading to eventual clearance (Pirmohamed et al., 1995). Therefore, all experiments that utilize CNO should verify that in the species and strain being employed, the administration of CNO does not have effects.

Recently, interest in CNO has increased due to its exploitation as a ligand for the engineered G protein-coupled receptors (GPCRs) in the chemogenetic Designer Receptors Exclusively Activated by Designer Drug (DREADD) system. Briefly, this system consists of a family of synthetic GPCRs (based upon the muscarinic M3 and M4 subtypes), which are not believed to be activated by any endogenous ligand but which are potently activated by the otherwise inert molecule CNO (Armbruster et al., 2007). This is an extension of a previous Receptor Activated Solely by a Synthetic Ligand (RASSL) concept (Coward et al., 1998; Redfern et al., 1999), with the key functional advantage being that, unlike the RASSL system, where the activating ligand has affinity for endogenous receptors within the CNS, in the DREADD system the ligand (CNO) is believed to be inert, that is, to have no biological activity. The ability to express DREADD receptors in neurons *in vivo* completes a system whereby the receptor, coupled to a downstream signaling cascade of choice [inhibition (Gi); depolarization and burst firing; increases in cAMP] is expressed in a neuronal subtype of interest (e.g., via stereotaxic infusion of virus particles), which can then be selectively and exclusively manipulated by the systemic administration of CNO (Farrell et al., 2013; Zhu et al., 2014). This is a potentially powerful research and therapeutic toolbox, which has already yielded novel insights into brain–behavior relationships. We chose to use this system to ask the following simple yet important question: can the behavioral effects we have previously seen following permanent lesions of a specific neuronal population also be induced by transient (DREADD-Gi) inhibition of the same neuronal population? Our initial results were somewhat surprising as we repeatedly observed that CNO-treated, non-DREADD, wild-type control rats were impaired in the behavior we predicted to be affected by DREADD-Gi inhibition. That is, in the absence of any DREADD receptor, we were observing behavioral effects of CNO. This was a major hurdle in interpreting our results. Therefore, before pursuing our DREADD experiments in alternative behaviors of interest, we undertook a series of control experiments, which are reported here. Using wild-type male Long–Evans rats, we investigated the effects of CNO at commonly used doses (1–5 mg/kg) on acoustic startle response (ASR), prepulse inhibition (PPI) of ASR, NMDA- and muscarinic-induced disruption of PPI, spontaneous locomotion, and amphetamine (AMPH)-induced hyperlocomotion. In addition, by use of fast-scan cyclic voltammetry (voltammetry), we assessed the effects of CNO on evoked dopamine (DA) release in the nucleus accumbens (NAcc). Finally, we analyzed plasma from CNO-treated rats by HPLC for detection of CNO, clozapine, and *N*-Des.

## Materials and Methods

Male Long–Evans rats (Harlan Laboratories; rats were bred in our facilities) weighing 300–330 g at the start of behavioral studies and 300–450 g for voltammetric studies were maintained single housed in plastic cages in a temperature- and humidity-controlled room. Lights were on a 12 h light/dark cycle (lights on at 7:00 A.M.) with testing conducted during the light phase. Rats had free access to food (Harlan Diet 2018, Harlan Laboratories) and water in the home cage. All experiments were approved by the Institutional Animal Care and Use Committee and conducted in accordance with the National Institutes of Health *Guide for the Care and Use of Laboratory Animals*.

### Compounds

CNO was supplied by the National Institutes of Health Drug Supply Program, and was dissolved in DMSO then diluted to a final concentration of 1, 2, or 5 mg/ml CNO in 0.5% DMSO in saline solution. Control injections were 0.5% DMSO in saline solution. Of the 22 articles that we found that used systemic administration of CNO in rats (Ferguson et al., 2011, 2013; Anderson et al., 2013; Michaelides et al., 2013; Boender et al., 2014; Bull et al., 2014; Dell’Anno et al., 2014; Kätzel et al., 2014; Robinson et al., 2014; Chang et al., 2015; Gompf et al., 2015; Mizoguchi et al., 2015; Pienaar et al., 2015; Scofield et al., 2015; Yau and McNally, 2015; Grace et al., 2016; Ma et al., 2016; Marchant et al., 2016; Qiu et al., 2016; Sengupta et al., 2016; Wicker and Forcelli, 2016; Wirtshafter and Stratford, 2016), 2 used chronic treatment (e.g., in drinking water) and 11 used doses >1 mg/kg. Of those that used doses of >1 mg/kg, most used doses of 3 mg/kg, but in a few cases doses were as high as 10 mg/kg. Of all the studies using rats and CNO-activated DREADDs that we surveyed, only a few used a non-DREADD CNO control, and most of these used this control in only a subset of the presented experiments. Our selection of 1, 2, and 5 mg/kg doses was based on our preliminary experience with CNO and the few published rat studies that were available at the time. Also, Dr. Roth’s research group had shown that in mice there were no effects at doses of 5 mg/kg (Alexander et al., 2009) and that some mouse studies had used doses as high as 10 mg/kg (Ray et al., 2011).

Phencyclidine (PCP; Sigma-Aldrich), d-amphetamine (Sigma-Aldrich), and scopolamine (Tocris Bioscience) were dissolved in 0.9% saline solution, and injected at 1 ml/kg.

### Behavioral testing

#### ASR and PPI testing

Testing was conducted in startle chambers (Kinder-Scientific). Each sound-attenuating test chamber was equipped with small chambers mounted on a parallelogram load cell (calibrated to newtons) situated directly beneath a loudspeaker. Constant dim illumination was provided by a light within the chamber. Throughout testing, constant background noise was presented as 65 dB of white noise. All startle and prepulses (PPs) were presented as squarewave (instantaneous rise and fall) bursts of white noise. A trial is defined as a startle stimuli (120 dB, 40 ms) preceded by either a PP (various conditions, described in a relevant subsection of Materials and Methods) or no prepulse (for assessment of startle only). The intertrial interval (ITI) is the time (in seconds) between trials, regardless of whether these trials contain a PP or not. The percentage PPI was calculated as [100 − (mean ASR amplitude on prepulse pulse trials)/mean 120 dB ASR amplitude on pulse alone trials) × 100]. All rats had an ASR >1 N on the 120 dB startle-only trials, and all rats were included in the analysis of PPI.

Following 5 min of acclimatization and the presentation of three 120 dB 40 ms pulses with a mean 15 s ITI, rats were exposed to eight trial types, presented 10 times each in a pseudorandom order with a mean ITI of 15 s (range, 5–25 s). Trial types were as follows: startle stimulus only (120, 110, 100, and 90 dB; 40 ms white noise) and four different prepulse plus pulse trials (68, 72, 76, and 80 dB prepulse, 20 ms duration, the onset of which was followed 120 ms later by a 120 db 40 ms pulse).

#### CNO-alone studies

To assess the effects of CNO on startle and PPI, rats were injected with CNO (1 mg/kg, i.p.) or 0.5% DMSO in saline solution 20 min before being placed in the PPI chambers. This dose was chosen based on the literature (Anderson et al., 2013; Michaelides et al., 2013) and consultation with investigators who had experience using DREADDs.

#### Phencyclidine studies

Knowing the possibility of CNO retroconversion, it was decided to test higher doses of CNO in a bioassay known to be sensitive to the presence of several antipsychotic agents, the disruption of prepulse inhibition by the NMDA antagonist PCP (Keith et al., 1991; Swerdlow et al., 1996). To assess the effects of CNO on PCP-induced disruption of PPI, rats were assigned to one of the following six experimental groups, each group containing two treatments: (1) vehicle (Veh) and vehicle; (2) vehicle and PCP; (3) CNO 2 mg/kg and vehicle; (4) CNO 5 mg/kg and vehicle; (5) CNO 2 mg/kg and PCP; and (6) CNO 5 mg/kg and PCP. Rats were injected with the first treatment (0.5% saline, i.p., in DMSO or CNO) followed 20 min later by the second treatment (saline or PCP 2.5 mg/kg, both s.c.). They were placed in the PPI chambers 10 min after the PCP injection. Due to there being no significant reversal of PCP-mediated disruption at CNO doses of 2 or 5 mg/kg, lower doses were not pursued.

#### Scopolamine studies

To assess the effects of CNO on scopolamine-induced disruption of PPI, rats were assigned to one of the following four groups, each containing two treatments: (1) vehicle and vehicle; (2) vehicle and scopolamine; (3) CNO 5 mg/kg and vehicle; and (4) CNO 5 mg/kg and scopolamine. Rats were injected with the first treatment (0.5% DMSO in saline or 5 mg/kg CNO, both i.p.) followed 20 min later by the second treatment (saline or 0.5 mg/kg scopolamine, both s.c.). They were placed in the PPI chambers 10 min after the scopolamine injection. In light of the results with PCP, initial studies used 5 mg/kg CNO, and, because no significant reversal was observed, further doses were not evaluated.

#### Locomotor testing

Locomotor testing was conducted in plastic cages measuring 45 × 23 × 20 cm interfaced by a grid array of infrared beams connected to a computer system that tracked and quantified the location and movements of the animal (OMNITECH Instruments). Rats were habituated to the testing room in their home cages for >30 min prior to testing. To guard against the possibility of a U-shaped dose–response curve, the effects of all three doses of CNO tested above were assessed for effects on spontaneous locomotion and amphetamine-induced hyperlocomotion. Rats were assigned to one of six groups, each containing two treatments. The groups were as follows: (1) vehicle and vehicle; (2) vehicle and amphetamine; (3) CNO 1 mg/kg and vehicle; (4) CNO 2 mg/kg and vehicle; (5) CNO 5 mg/kg and vehicle; (6) CNO 1 mg/kg and amphetamine; (7) CNO 2 mg/kg and amphetamine; and (8) CNO 5 mg/kg and amphetamine. Rats were injected with the first treatment (0.5% DMSO in saline or CNO, both i.p.) and immediately placed in the locomotor cages; 20 min later, they were removed from the cages and injected with the second treatment (saline or 1.5 mg/kg d-amphetamine, both s.c.), and placed back in the locomotor cages for a further 120 min.

### Fast-Scan Cyclic Voltammetry

Rats were anesthetized with urethane (1.5–2.0 g/kg), placed into a stereotaxic apparatus, and implanted with a carbon fiber microelectrode aimed at the NAcc [+1.3 anterior, +1.3 lateral (L), −6.5 ventral (V), relative to bregma] and an Ag/AgCl reference electrode located in the contralateral cortex (España et al., 2010, 2011). A bipolar stimulating electrode (Plastics One) aimed at the ventral tegmental area (−5.2 posterior, +1.1 L, −7 V) was lowered in 100–200 µm increments until a 1.0 s, 60 Hz monophasic (4 ms; 700 µA) stimulation train produced a robust DA response in the NAcc. Stimulation evoked DA release was recorded every 5 min for at least 30 min until DA peaks in the NAcc reached stability (three consecutive collections within 10%). Once stability was achieved, rats were injected intraperitoneally with vehicle (0.5% DMSO in 0.9% normal saline) and ensuing changes in DA release were recorded for at least 30 min until DA peak height in the NAcc reached stability. Due to the results of the locomotor studies, we focused on the two higher doses of CNO (2 and 5 mg/kg) for the neurochemical studies, where we also used amphetamine. Rats were then injected intraperitoneally with either a second dose of vehicle (volume equivalent to a 5 mg/kg dose of CNO), 2 mg/kg CNO, or 5 mg/kg CNO, and changes in DA release and uptake (tau) were monitored for 1 h, at which point they were injected with AMPH (1.5 mg/kg). DA release and uptake were determined at 30 min following each treatment and expressed as a percentage of baseline (i.e., the average of the last three collections prior to treatment). The effect of CNO on DA release and uptake for each animal was calculated as (DA_CNO_/DA_Pre-CNO Baseline_)/(DA_Vehicle_/DA_Pre-Vehicle Baseline_) to control for any effects of the vehicle itself on DA signaling. The effect of AMPH on DA release and uptake in each animal was calculated as (DA_AMPH_/DA_Pre-AMPH Baseline_).

### Data acquisition

The electrode potential was linearly scanned (0.4–1.2 V and back to −0.4 V vs Ag/AgCl), and cyclic voltammograms were recorded at the carbon fiber electrode every 100 ms with a scan rate of 400 V/s using a voltammeter/amperometer (Chem-Clamp, Dagan Corporation). The magnitude of stimulated DA release and transporter-mediated uptake kinetics was monitored. DA overflow curves were analyzed, as previously described for peak concentrations of DA and *tau*, using Demon Voltammetry and Analysis software written in LabVIEW language (National Instruments; Yorgason et al., 2011).

### HPLC studies

Clozapine (assay 98.9%) was obtained from MP Biomedicals. *N*-desmethylclozapine (assay >99%) and doxipine HCL internal standard (assay >99%) were obtained from Tocris Bioscience. For these studies, only CNO 5 mg/kg was tested because it was efficacious in the voltammetry and locomotor studies. In addition, CNO at 1 mg/kg had been previously shown to retroconvert to clozapine in mice (Guettier et al., 2009). Rats were administered CNO 5 mg/kg, i.p., and 30, 90, 180, or 360 min later (*n* = 4–5/time point) were deeply anaesthetized with sodium pentobarbital (100 mg/kg, i.p.; Fatal Plus, Vortech Pharmaceuticals Ltd.), the heart was exposed, and 8–10 ml of blood was drawn from the right ventricle into a syringe containing EDTA (final concentration, 4 mm). Blood was transferred to a chilled centrifuge tube and spun at 1000 × *g* at 5°C for 10 min. Plasma was separated into 1 ml aliquots, the HPLC internal standard (doxepin HCL, 20 µl of a 1 µm solution) was added, and then aliquots were stored at −80^°^C until further processing. Control plasma, for use in matrix-based calibrators, interference, recovery, and limit of quantification measurements, was collected in the same manner from rats not treated with CNO.

### Solid-phase extraction

C2 extraction columns (100 mg/ml; ISOLUTE column, Biotage) were conditioned by sequential washing with 0.5 ml of elution solution (10 mm acetic acid, 5 mm trimethylamine), 3 × 1 ml methanol and 2 × 1 ml buffer solution rinses (100 mm sodium phosphate dihydrate, pH 4.6). Plasma (900 µl) was loaded to the columns and allowed to flow under gravity. Columns were then washed sequentially with 2 × 1 ml water and 2 × 0.5 ml acetonitrile, and vacuum dried for 5 min. The analytes were eluted with a 2 × 0.5 ml elution solution, and the combined eluate was evaporated at 30°C under nitrogen. The dry residue was reconstituted in 500 µl of 0.1 m HCL. The extraction recovery of clozapine, *N*-desmethylclozapine, clozapine N-oxide, and doxepin (internal standard) were between 85% and 95%.

### HPLC

HPLC analysis was performed by a modification of the method described by Mosier et al. (2003) on a Shimadzu Scientific Instruments Prominence 20A series HPLC and SPDM20A photodiode array. Stock solutions were prepared in 0.1 m HCl, and calibrators were prepared using blank plasma-matrix. Calibrators were subjected to solid-phase extraction (SPE), as described above for samples. Quantitation was performed by an internal standard methodology, using doxepin as the internal standard. SPE extracts (50 µl) were injected into a 250 × 4.6 mm SupelcoSil, LC-CN (cyano), 5 µm analytical column with a 2 cm guard column, and eluted isocratically with acetonitrile/80 mm ammonium acetate (pH 7; 75:25 v/v) at a flow rate of 1.2 ml/min. Photodiode array detection was at 254 nm using a 4 nm bandwidth. Matrix and interference studies demonstrated a lack of any coeluting peaks with target analyte peaks. Blank plasma sample chromatograms were subtracted from each sample and calibrator chromatogram to minimize fluctuations in the background chromatogram. The quantitation limits for clozapine, clozapine N-oxide, and *N*-desmethylclozapine were 0.01, 0.03, and 0.03 μm, respectively. Quantitative spiking studies using CLZ, *N*-Des, and CNO demonstrated 95–102% recovery of a 0.1 µm standard addition. The between-run % coefficient of variation was <5% for each analyte. Analysis of blank plasma spiked with CNO found a reduction of CNO to clozapine during sample and HPLC processing, which occurs during some processing methods (Lin et al., 1994), to be <2.5%.

### Data analysis

Behavioral and neurochemical data were analyzed in SPSS version 22 (IBM). Details of individual tests are described within the relevant section in Results. Where graphs are displayed, these depict group means ± SEM. Results were considered statistically significant when *p* < 0.05. On graphs, * indicates significant difference at the *p* = 0.01–0.05 confidence level, and ** indicates *p* ≤ 0.01.

## Results

### Behavioral studies

#### Effects of CNO on startle and PPI

**Figure 1.**
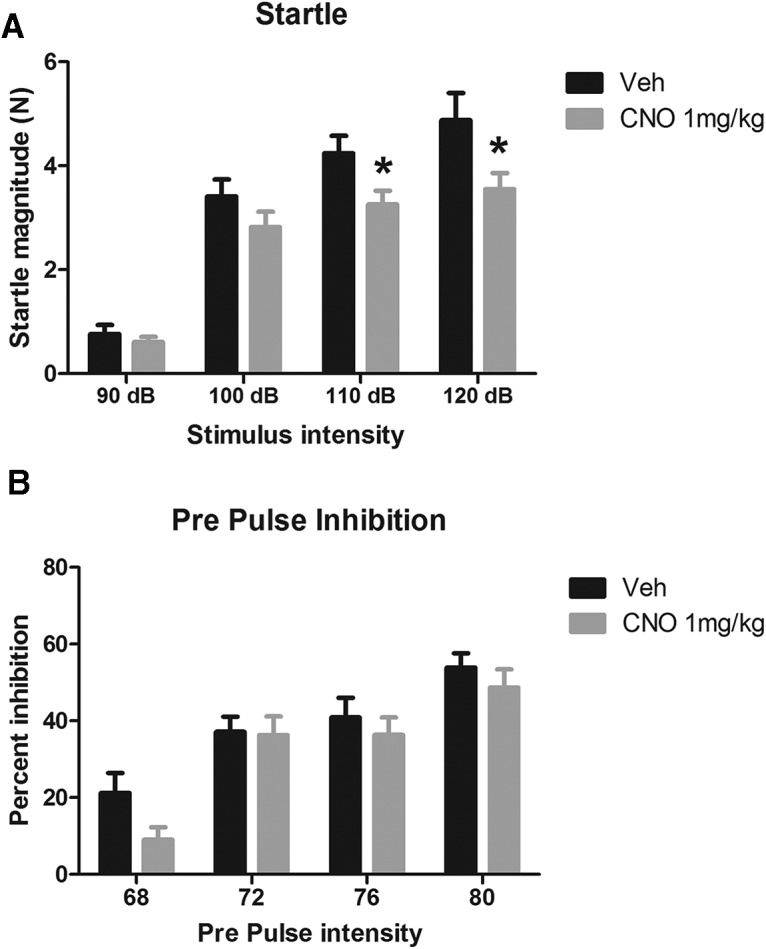
***A***, ***B***, Startle magnitude and PPI after treatment with CNO. CNO significantly reduced the startle response to 110 and 120 dB startle stimuli (***A***), but had no significant effect on PPI (***B***). *p < 0.05.

Results showing the effects of CNO on the ASR and PPI are shown in Figure 1*A*. CNO significantly reduced the ASR to 110 and 120 dB stimuli, but not to stimuli of lower intensities (Fig. 1*A*). CNO had no effect on PPI at any PP level tested (Fig. 1*B*). The effects of CNO on the ASR were analyzed with repeated-measures ANOVA, which showed a significant effect of startle dB (*F*_(3,90)_ = 124.3; p <0.001), drug treatment (*F*_(1,30)_ = 4.31; *p* = 0.047), and a startle decibel × drug treatment interaction (*F*_(3,90)_ = 3.20; *p* = 0.027). The interaction was investigated with Sidak-adjusted pairwise comparisons, which found that at the 120 and 110 dB level CNO-treated rats had a significantly lower ASR than Veh-treated rats (*p* = 0.037 and *p* = 0.028), but there was no difference at the lower intensities (100 dB, *p* = 0.188; 90 dB, *p* = 0.440). The effects of CNO on PPI were also investigated with a repeated-measures ANOVA, which showed a main effect of PP intensity (*F*_(3,90)_ = 42.04; *p* < 0.001), no PP × drug treatment interaction (*p* = 0.374), and no main effect of drug (*p* = 0.256). As none of the effects involving CNO (drug treatment) were significant, no further analysis was performed on the PPI data (*n* = 16/group). Together, these results show that CNO at 1 mg/kg significantly reduced the ASR to 120 and 110 dB startling stimuli, but had no significant effect on PPI.

#### Effects of CNO on PCP-induced disruptions of ASR and PPI

**Figure 2.**
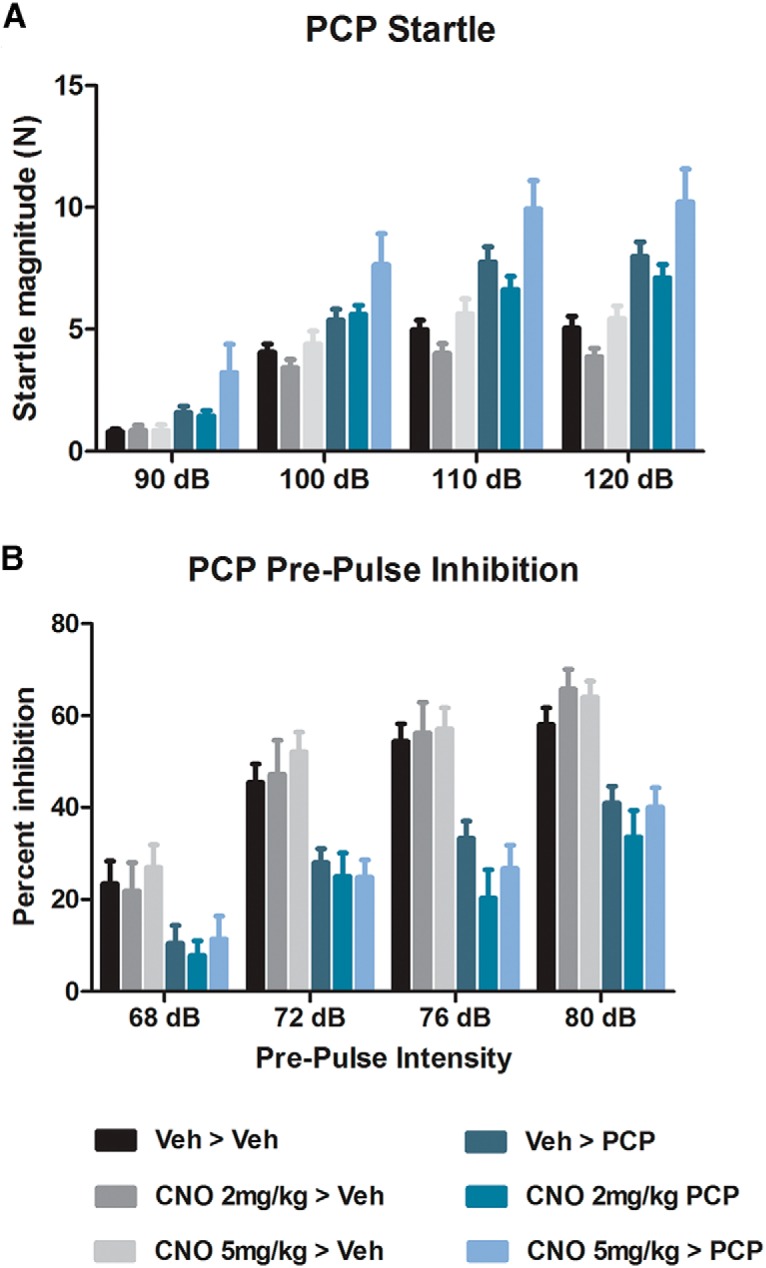
Startle magnitude and PPI after treatment with CNO and PCP. ***A***, ***B***, PCP significantly increased the startle magnitude (***A***) and disrupted the PPI (***B***). CNO pretreatment had no effect on the disruptive effects of PCP.

Results showing the effects of 2 and 5 mg/kg CNO on PCP-induced disruptions of the ASR and PPI are shown in Figure 2. Pretreatment with CNO had no effect on the PCP-induced increase in the ASR (Fig. 2*A*) or PCP-induced decrease in PPI (Fig. 2*B*). The effects on the ASR were investigated with a two-way repeated-measures ANOVA, which found a main effect of dB (*F*_(3,219)_ = 427.14; *p* < 0.001) and PCP treatment (*F*_(1,73)_ = 32.517; *p* < 0.001), and a decibel × PCP treatment interaction (*F*_(3,219)_ = 3.23; *p* = 0.005), but both the CNO treatment × PCP treatment (*F*_(2,73)_ = 1.376; *p* = 0.259) and dB × PCP treatment × CNO treatment (*F*_(6,219)_ = 0.433; *p* = 0.856) interactions were nonsignificant. Sidak test-adjusted pairwise comparisons investigating the effect of PCP treatment found that PCP increased the ASR at all stimuli intensities (90 dB, *p* = 0.001; 100 dB, *p* < 0.001; 110 dB, *p* < 0.001; 120 dB, *p* < 0.001). As no interactions involving CNO were significant, no further analyses were performed. The effects of PCP and CNO on PPI were also investigated with a two-way repeated-measures ANOVA, which showed a main effect of PP intensity (*F*_(3,222)_ = 119.98; *p* < 0.001) and PCP treatment (*F*_(1,74)_ = 50.90; *p* < 0.001), and a PP × PCP treatment interaction (*F*_(3,222)_ = 5.89; *p* = 0.001), but that both the CNO treatment × PCP treatment (*F*_(2,74)_ = 0.859; *p* = 0.428) and PP × PCP treatment × CNO treatment (*F*_(6,222)_ = 0.827; *p* = 0.550) interactions were nonsignificant. Sidak-adjusted pairwise comparisons investigating the effect of PCP treatment found that PCP decreased PPI at all PP levels (68 PP, *p* = 0.001; 72 PP, *p* < 0.001; 76 PP, *p* < 0.001; 80 PP, *p* < 0.001). As no interactions involving CNO were significant, no further analyses were performed (*n* = 11–18/group). Combined, these results show that PCP treatment significantly increased the ASR and significantly decreased PPI, and that pretreatment with CNO (at either 2 or 5 mg/kg) had no effect on the changes caused by PCP.

#### Effects of CNO on scopolamine-induced disruptions of ASR and PPI

**Figure 3.**
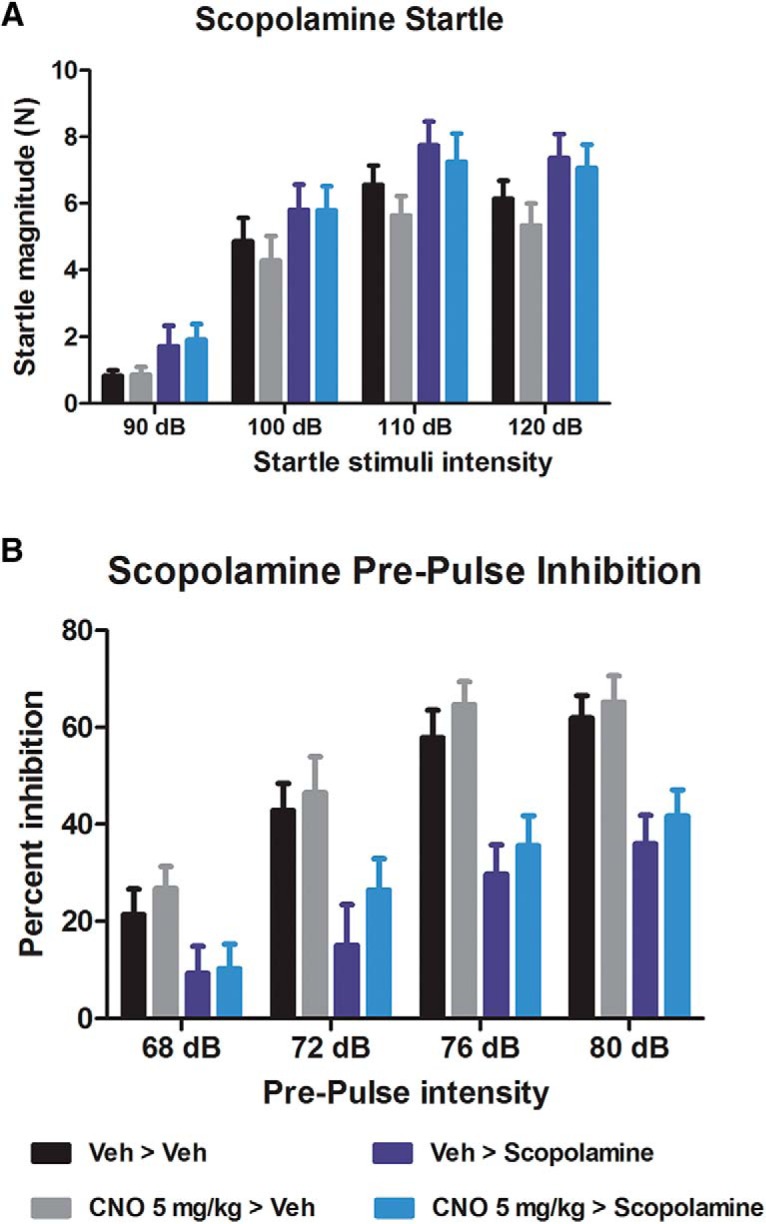
Startle magnitude and PPI after treatment with CNO and scopolamine. ***A***, ***B***, Scopolamine significantly increased the startle magnitude (***A***) and disrupted the PPI (***B***). CNO pretreatment had no effect on the disruptive effects of scopolamine.

Results showing the effects of 5 mg/kg CNO on scopolamine-induced disruptions of the ASR and PPI are shown in Figure 3. Pretreatment with CNO had no effect on scopolamine-induced increase in the ASR (Fig. 3*A*) or scopolamine-induced decrease in PPI (Fig. 3*B*). Effects on the ASR were investigated with a two-way repeated-measures ANOVA, which found a main effect of decibel (*F*_(3,84)_ = 248.50; *p* < 0.001) and scopolamine treatment (*F*_(1,28)_ = 4.874; *p* = 0.036), but that the dB × scopolamine treatment interaction (*F*_(3,84)_ = 0.485; *p* = 0.694) and both the CNO treatment × scopolamine treatment (*F*_(1,28)_ = 0.127; *p* = 0.725) and decibel × scopolamine treatment × CNO treatment (*F*_(3,84)_ = 0.081; *p* = 0.970) interactions were nonsignificant. Sidak-adjusted pairwise comparisons investigating the effect of scopolamine treatment found that scopolamine increased the ASR at all decibel levels (90 dB, *p* = 0.01; 100 dB, *p* = 0.002; 110 dB, *p* < 0.001; 120 dB, *p* = <0.001). As no interactions involving CNO were significant, no further analyses were performed. Effects of scopolamine and CNO on PPI were also investigated with a two-way repeated-measures ANOVA, which showed a main effect of PP intensity (*F*_(3,84)_ = 99.86; *p* < 0.001) and scopolamine treatment (*F*_(1,28)_ = 19.58; p <0.001), and a PP × scopolamine treatment interaction (*F*_(3,84)_ = 3.851; *p* = 0.012), but that both the CNO treatment × scopolamine treatment (*F*_(1,28)_ = 0.014; *p* = 0.907) and PP × scopolamine treatment × CNO treatment (*F*_(3,84)_ = 0.689; *p* = 0.561) interactions were nonsignificant. Sidak-adjusted pairwise comparisons investigating the effect of scopolamine treatment found that scopolamine decreased PPI at all PP levels (68 PP, *p* = 0.01; 72 PP, *p* = 0.002; 76 PP, *p* < 0.001; 80 PP, *p* < 0.001; *n* = 8/group). As no interactions involving CNO were significant, no further analyses were performed. Combined, these results show that scopolamine treatment significantly increased the ASR and significantly decreased PPI, and that pretreatment with CNO had no effect on the changes caused by scopolamine.

#### Effects of CNO on spontaneous locomotion and amphetamine-induced hyperlocomotion

**Figure 4.**
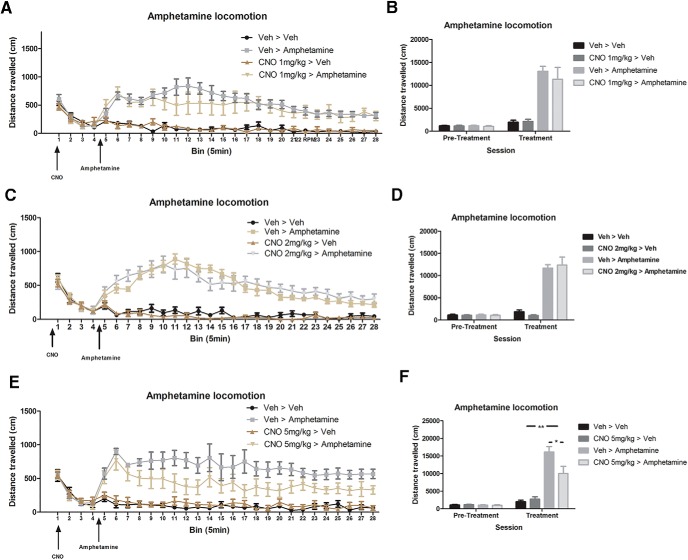
Effects of CNO on spontaneous locomotion and amphetamine-induced hyperlocomotion. Rats were pretreated with either vehicle or CNO, followed 20 min later by either vehicle or amphetamine. ***A–F***, CNO at 1 mg/kg (***A***, ***B***), 2 mg/kg (***C***, ***D***), or 5 mg/kg (***E***, ***F***) had no effect on spontaneous locomotion. Neither 1 nor 2 mg/kg CNO altered amphetamine-induced hyperlocomotion (***B***, ***D***), but 5 mg/kg CNO significantly reduced the effects of amphetamine (***F***). *p < 0.05, **p < 0.01.

The effect of CNO on both spontaneous and amphetamine-induced locomotion was determined at the doses of 1, 2, and 5 mg/kg (i.p.). CNO at 1 and 2 mg/kg had no effect on spontaneous locomotion or amphetamine-induced hyperlocomotion (Fig. 4*A–D*). Analyses were performed on the total movement (distance traveled) during the 20 min CNO pretreatment (Veh or CNO) and 120 min post-treatment (Veh or amphetamine) by two-way ANOVA (for all groups, *n* = 8). Sidak-corrected pairwise comparisons found that during the 120 min amphetamine testing period, all amphetamine-treated groups moved significantly more than all non-amphetamine-treated groups (*p* < 0.001 in all cases). As no main effects of CNO treatment or interactions involving CNO treatment were significant, no further analyses were performed. For all groups, *n* = 8.

The effects of 5 mg/kg CNO on spontaneous locomotion and amphetamine-induced hyperlocomotion are shown in Figure 4, *E* and *F*. Two-way ANOVA finds no significant differences for the total movement during the 20 min CNO pretreatment period [no effect of CNO treatment (*F*_(1,32)_ = 0.16; *p* = 0.901) and no pre-existing effect in the amphetamine groups or interactions involving amphetamine treatment: amphetamine treatment (*F*_(1,32)_ = 0.476; *p* = 0.496), CNO treatment × amphetamine treatment (*F*_(1,32)_ = 0.205; *p* = 0.654)]. For the 120 min period after the second treatment, two-way ANOVA finds a main effect of amphetamine treatment (*F*_(1,32)_ = 63.791; *p* < 0.001), a nearly significant effect of CNO treatment (*F*_(1,32)_ = 4.027; *p* = 0.055), and a significant CNO treatment × amphetamine treatment interaction (*F*_(1,32)_ = 6.489; *p* = 0.017). Sidak-adjusted pairwise comparisons investigating the interaction found no significant difference between the vehicle plus vehicle group and the CNO 5 mg/kg plus vehicle group (*p* = 0.999), and a significant increase in movement in vehicle plus amphetamine group (*p* < 0.001), and that CNO plus amphetamine group moved significantly more than the saline plus saline group (*p* = 0.001) but significantly less than the saline plus amphetamine group (*p* = 0.019). Together, these results show that CNO at a dose of 5 mg/kg has no effect by itself on spontaneous locomotion, but significantly reduced the hyperlocomotion caused by amphetamine treatment. In order to try and establish the time course of the reduction in amphetamine hyperlocomotion caused by CNO pretreatment, a repeated-measures ANOVA was performed on the locomotor data grouped into 5 min bins (Fig. 4*A*,*C*,*E*). There was a main effect of amphetamine treatment (*F*_(1,28)_ = 63.791; *p* < 0.001), bin (*F*_(23,644)_ = 3.453; *p* < 0.001), a bin × treatment interaction (*F*_(23,644)_ = 1.804; *p* = 0.012), and a CNO treatment × amphetamine treatment interaction (*F*_(1,28)_ = 6.489; *p* = 0.017). Sidak-corrected pairwise comparisons performed on each bin found that 6 of the CNO plus amphetamine bins were lower than the saline plus amphetamine bins (bins 8, 13, 16, 20, 22, and 24, *p* < 0.05 in all cases) and 13 of the CNO plus amphetamine bins are not significantly different from the vehicle plus vehicle bins (5, 7, 8, 9, 10, 11, 12, 13, 14, 15, 16, 21, and 23, *p* > 0.05 in all cases). The relatively equal distribution of these effects across the 120 min session and lack of a bin × CNO treatment × amphetamine treatment interaction in the repeated-measures ANOVA (*F*_(23,644)_ = 0.671; *p* = 0.876) suggest that the effect of CNO on amphetamine treatment was equal throughout the session rather than, for example, evident only early or late in the session. Together, these results show that CNO at a dose of 5 mg/kg had no significant effect on spontaneous locomotion, but significantly reduced amphetamine-induced hyperlocomotion.

### Electrochemical recordings

**Figure 5.**
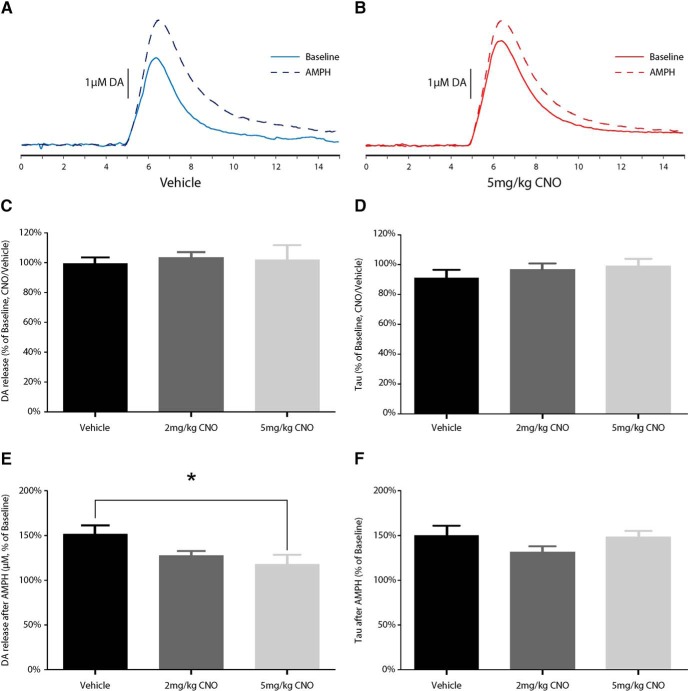
Effects of CNO on baseline and amphetamine-induced DA signaling. ***A***, ***B***, Examples of 15 s DA overflow curves in vehicle-treated (***A***) and CNO-treated (***B***) rats before (solid) and after (dashed) a dose of 1.5 mg/kg, i.p., d-AMPH. ***C***, ***D***, CNO did not alter stimulated DA release (***C***) or uptake (***D***) under baseline conditions. ***E***, ***F***, CNO dose-dependently blunted the increased DA release in response to d-AMPH 30 min after systemic treatment (***E***); however, there were no significant effects on the magnitude of DA uptake inhibition (***F***). *p < 0.05

The effects of CNO on DA release and uptake are shown in Figure 5. CNO alone did not significantly affect electrically stimulated DA release in the NAcc, as determined by one-way ANOVA (Welch’s ANOVA for unequal variances, *F*_(2,9.269)_ = 0.221, *p* = 0.805), nor was there an effect on DA uptake (*F*_(2,15)_ = 0.685, *p* = 0.519). By contrast, CNO significantly decreased the effects of AMPH-induced increases in DA release 30 min following administration of AMPH, as determined by one-way ANOVA (*F*_(2,15)_ = 3.935, *p* < 0.05). *Post hoc* analysis using Bonferroni comparisons found that these effects reached significance only at 5 mg/kg (*p* < 0.05). Animals treated with 2 mg/kg CNO did not significantly differ from either vehicle-treated (*p* = 0.215) or 5 mg/kg CNO-treated (*p* = 1.000) animals. There was no effect of CNO on DA uptake, as determined by one-way ANOVA (tau; *F*_(2,15)_ = 1.664, *p* = 0.223).

### HPLC results

**Figure 6.**
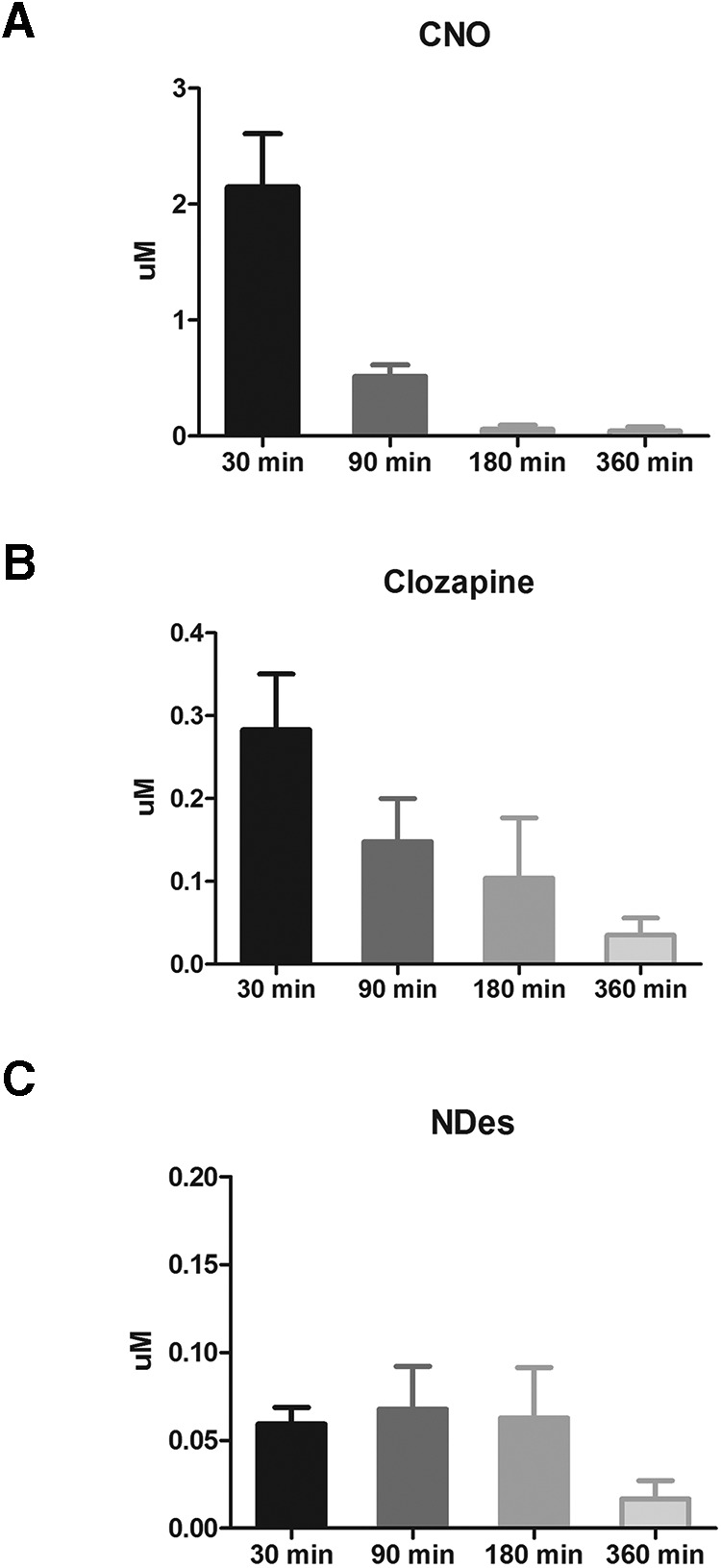
***A–C***, Plasma levels of CNO (***A***), clozapine (***B***), and *N*-Des (***C***) at various time points after the administration of 5 mg/kg CNO.

CNO levels were maximal (2.148 µm) at the 30 min collection time point and steadily diminished to very low levels (0.045 µm) at the 360 min collection time point (Fig. 6*A*). This is in line with the relatively rapid clearance of CNO in rodents (Baldessarini et al., 1993; Guettier et al., 2009) compared with humans (Jann et al., 1993). Clozapine was detectable at all time points and followed the same pattern as CNO—highest at the 30 min collection time (0.283 µm) and then diminishing as a function of time (Fig. 6*B*). Levels of *N*-Des followed a different pattern, remaining at a reasonably steady level (0.059–0.063 µm) until diminishing at the 360 min time point (Fig. 6*C*).

## Discussion

These experiments were conducted to investigate the effects of CNO in male Long–Evans rats. We assessed whether CNO has any effect on acoustic startle (Fig. 1*A*), PPI (Fig. 1*B*), and spontaneous locomotion (Fig. 4). We then assessed whether CNO modifies NMDA (PCP) and muscarinic (scopolamine)-induced disruption of PPI (Figs. 2, 3), and whether CNO can reduce amphetamine-induced hyperlocomotion (Fig. 4). In order to assess the neurochemical effects, we performed voltammetry in the NAcc to measure the effects of CNO on electrically evoked DA release alone and in response to amphetamine (Fig. 5*A*,*B*). We then processed plasma from CNO-treated rats in order to assess whether CNO is reduced to clozapine or converted into *N*-Des (Fig. 6*A–C*). Our results show that the administration of CNO has multiple effects *in vivo*, and is converted to both clozapine and *N*-Des.

In the startle and PPI experiments, we found that 1 mg/kg CNO reduced the startle response to loud acoustic stimuli (Fig. 1) but had no effect on PPI (a measure of sensorimotor gating; for review, see Swerdlow et al., 2008). CNO (2 and 5 mg/kg) had no effect on the disruption to PPI induced by the NMDA antagonist PCP (Fig. 2) or the muscarinic antagonist scopolamine (Fig. 3). Because the higher doses of CNO had no effect on these measures, lower doses were not tested in PPI. In the locomotor studies, CNO alone (at 1 and 2 mg/kg) had no effect on spontaneous or amphetamine-induced locomotion (Fig. 4), but 5 mg/kg CNO pretreatment significantly attenuated amphetamine-induced hyperlocomotion (Fig. 4*F*). Combined, these results show that the administration of CNO within the dose range of 1–5 mg/kg has behavioral effects in Long–Evans rats. The electrochemical experiments mirror these results: although CNO treatment alone did not alter electrically evoked DA release, 5 mg/kg CNO significantly attenuated the increase in evoked DA in response to systemic d-amphetamine.

In order to establish whether it is possible that our results could be due to the conversion of CNO into clozapine or *N*-Des (as has been reported previously by Lin et al., 1996), we processed plasma from rats treated with CNO for the HPLC detection of CNO, clozapine, and *N*-Des. After the administration of 5 mg/kg CNO, plasma CNO levels peaked quickly and fell to very low levels within 360 min (Fig. 6*A*). Clozapine was detectable at all time points (Fig. 6*B*), again with the maximum concentration at 30 min (clozapine concentration was ∼13% of CNO) and fell steadily across the later time points. The level of clozapine at 30 min was approximately one-tenth of the level of CNO at the same time point. A similar ratio has been seen after CNO administration in rats (Lin et al., 1996) and humans (Jann et al., 1994; Chang et al., 1998). Most notably, even though no values were provided, the figure provided by Guettier et al. (2009) shows what appears to be a similar CNO/clozapine ratio and time course in mice at 30 min after the administration of 1 mg/kg CNO (Guettier et al., 2009). The authors summarized their results by stating that the levels of clozapine produced were nonsignificant. For their experimental manipulation, they are correct in this assumption, as CNO did not produce noticeable effects in a wild-type control group. In our studies, low levels of *N*-Des were also detected at all time points (Fig. 6*C*), but in a seemingly different pattern than the clozapine, staying relatively steady until the 360 min time point. These results are in line with previous reports showing the rapid clearance of CNO in rodents (Baldessarini et al., 1993; Guettier et al., 2009), and the conversion of CNO to clozapine and *N*-Des in rats (Lin et al., 1996). Given that in our studies the levels of *N*-Des were always lower than those of either CNO or clozapine, and remained at a stable level until both CNO and clozapine were almost completely absent (360 min), one plausible explanation is that a portion of the CNO is reduced to clozapine, which is then metabolized into *N*-Des (Lin et al., 1996; Mosier et al., 2003).

The detected levels of peripheral plasma clozapine in our rats are comparable to the ranges of clozapine known to have behavioral effects. In rats, 10 mg/kg clozapine (s.c.) leads to a peak unbound plasma level from intracranial microdialysis sampling of ∼0.07 µm, and a delayed peak level of 0.008 µm *N*-Des (Cremers et al., 2012). At this dose, clozapine has been shown to reduce amphetamine hyperlocomotion (Natesan et al., 2007) and reduces the ASR in rats (Conti et al., 2005; Feifel et al., 2011), just as we observed after CNO administration. However, a direct comparison of dose responses to these compounds in the literature is lacking. On the contrary, we did not observe a reversal of the PPI disruptive effects of PCP or scopolamine with CNO administration, as would be expected if clozapine is the main biologically active compound in circulation after CNO administration. However, it would be rash to then conclude that CNO is pharmacologically responsible for the effects we have observed. It has to be considered that when clozapine is administered, CNO is created but not in as great quantities or in the same ratio as we have in the current situation of administering CNO. The presence of CNO may impact the pharmacological action of both clozapine and *N*-Des by simply altering the metabolism, clearance, distribution, and ultimately the time course of action. To attempt to tease this apart with the administration of clozapine or *N*-Des, which themselves will then produce CNO and each other (albeit at different levels), is something that would require a tour de force effort that combines plasma analysis from the same animals that perform the behavior and the time courses of drug metabolism. Therefore, the simplest and most relatable data that should be gathered are a within-study comparison of the effects of CNO in control animals, regardless of the purported known metabolism in any one species. Moreover, both clozapine and *N*-Des have a complex pharmacology. Clozapine is a potent antipsychotic agent, which has submicromolar affinity for >25 receptors within the CNS, principally serotonergic, muscarinic, dopaminergic, noradrenergic, and histaminergic receptors (Coward, 1992; Schotte et al., 1993; Peters, 2012). *N*-Des also interacts with multiple systems within the CNS but has a notably different pharmacology than clozapine (e.g., muscarinic M1 receptor; Sur et al., 2003; Davies et al., 2005; Thomas et al., 2010). The additive or synergistic effects of even undetectable levels of these compounds cannot be assessed or speculated for every circumstance or biological question, especially since the clearance of the compounds will be complicated with the presence of CNO and so needs to be empirically evaluated. In conclusion, despite CNO levels being much higher than the clozapine levels, we cannot make a firm conclusion as to the compound responsible for the behavioral effects after CNO administration.

It is important to emphasize that from the presented results it cannot be unequivocally determined which of the three compounds (CNO, clozapine, or *N*-Des) are responsible for the effects in our study. Further, it would be highly speculative and imprudent for us to suggest that a “safe” or inert dose of CNO can be extrapolated from our experiments. But rather, laboratories using CNO should validate its use in the species, strain, and paradigms being used, and also be aware that an effect of “CNO only” can be unmasked when the system is challenged, as per our amphetamine data (Fig 4). At minimum, our data highlight the need for experiments using CNO to include a CNO-treated control group devoid of DREADD receptors.

## Relevance to the DREADD system: is CNO an inert ligand?

The results from these studies show that CNO, in male Long–Evans rats, is not an inert ligand. Regardless of whether the behavioral effects we observed are due to CNO itself or to the conversion to clozapine and *N*-Des, the administration of CNO is not without consequence. However, perhaps the biggest concern is indeed the conversion of CNO into clozapine and *N*-Des. We did not perform HPLC analysis on the lower doses of CNO used in the behavioral studies because it is assumed that those doses of CNO would be metabolized in a similar manner and ratio. Moreover, if we were to perform the study with 1 mg/kg CNO but failed to detect clozapine and *N*-Des by our HPLC assay, this could indicate that levels are simply below our detection limits for clozapine and *N*-Des of 0.01 and 0.03 μm, respectively. However, this study has been performed in mice, with low but detectable levels of clozapine being detected by liquid chromatography-tandem mass spectrometry after the administration of 1 mg/kg CNO (Guettier et al., 2009). In combination with a control group that was devoid of DREADD receptors, Guettier et al. (2009) rightly conclude that for their paradigm the level of clozapine was not significant. Combined, the effects of CNO administration on the ASR (Fig. 1), attenuation of the effects of amphetamine (Figs. 4, 5) without an effect on spontaneous locomotion in the absence of amphetamine (Fig. 4), and the presence of clozapine and *N*-Des in the plasma (Fig. 6) means that the administration of CNO has no single and clearly predictable effect on one system, but instead is likely to have numerous effects on a diffuse range of systems.

Despite the issues raised, the present findings do not render CNO unusable as an activating ligand in the DREADD system. Rather, it highlights the necessity for incorporation of the appropriate controls and careful consideration of the doses to be administered. An experimental design that includes a group of animals that do not express DREADD receptors, but are given the same dose of CNO as the DREADD-expressing animals, seems a logical and necessary control. However, only a small minority of the current DREADD studies use this control. Instead, most prefer to use a within-subjects design where, in DREADD-expressing animals, the response to a vehicle injection is compared with the response to a CNO injection. In this design, it is impossible to separate out the effects of activation of the DREADD from any unexpected effects of CNO, which could be the enhancement or blockade of the expected result of DREADD activation. In terms of dosing, a wide range of doses (0.2–10 mg/kg) is regularly used in DREADD experiments (Alexander et al., 2009; Ferguson et al., 2011, 2013; Ray et al., 2011; Agulhon et al., 2013; Anderson et al., 2013; Farrell et al., 2013; Michaelides et al., 2013; Wang et al., 2013; Boender et al., 2014; Bull et al., 2014; Dell’Anno et al., 2014; Kätzel et al., 2014; Robinson et al., 2014; Zhu et al., 2014; Chang et al., 2015; Gompf et al., 2015; Mizoguchi et al., 2015; Pienaar et al., 2015; Scofield et al., 2015; Yau and McNally, 2015; Grace et al., 2016; Ma et al., 2016; Marchant et al., 2016; Qiu et al., 2016; Sengupta et al., 2016; Wicker and Forcelli, 2016), and there is seldom any explanation given as to how the dose that was used was decided upon. Using the lowest effectual dose in the assay to be performed, that which in the non-DREADD-expressing animals is experimentally silent, would seem the most straightforward way to minimize any off-target effects of CNO. Finally, perhaps of greatest concern, is the long-term administration of CNO, either in the drinking water or via minipump implantation. Long-term administration of clozapine at doses as low as 1.5 mg/kg/d has been shown to have diverse effects, including reducing 5-HT(2A) receptor mRNA in the striatum, accumbens, and hippocampus (Huang et al., 2007).

Our experiments were conducted in adult male Long–Evans rats, which, in addition to being a common outbred strain used in many behavioral experiments, are also the genetic background of the pTH:cre and pChAT:cre rats developed by Witten et al. (2011). The generalization of our metabolism results to other rat strains, or to mice, should be done with caution. This is because there are differences in the metabolism of clozapine between species; for example, humans convert considerably more clozapine to *N*-Des than do rats (Lin et al., 1996; Bun et al., 1999), and the elimination half-life of clozapine is markedly shorter in rats than humans (Jann et al., 1993) and also varies according to sex (Bun et al., 1999). However, the implications of our results are clear and generalizable: when conducting experiments with CNO, the consideration of possible biological effects of CNO administration must be taken into account. Encouragingly, analogs of CNO that activate DREADDs *in vitro* have been developed (Chen et al., 2015), and it is possible that one of these, or a future compound, will not be metabolized into clozapine-related compounds and will have fewer off-target effects than CNO.

## Synthesis

The decision was a result of the Reviewing Editor Karen Szumlinski and the peer reviewers coming together and discussing their recommendations until a consensus was reached. A fact-based synthesis statement explaining their decision and outlining what is needed to prepare a revision is listed below. The following reviewer(s) agreed to reveal their identity: Susan Ferguson

After consultation with the two experts reviewers of the revised report, I am happy to accept your article for publication in eNeuro, pending a minor revision. I do request prior to full acceptance that the authors make a more concerted effort in the Methods section to explain why the specific CNO dose(s) are employed within each experiment. These explanations can be brief. We all appreciate the authors efforts in revising the manuscript.

## References

[B1] Agulhon C, Boyt KM, Xie AX, Friocourt F, Roth BL, McCarthy KD (2013) Modulation of the autonomic nervous system and behaviour by acute glial cell Gq protein-coupled receptor activation in vivo. J Physiol 591:5599–5609. 10.1113/jphysiol.2013.261289 24042499 PMC3853498

[B2] Alexander GM, Rogan SC, Abbas AI, Armbruster BN, Pei Y, Allen JA, Nonneman RJ, Hartmann J, Moy SS, Nicolelis MA, McNamara JO, Roth BL (2009) Remote control of neuronal activity in transgenic mice expressing evolved G protein-coupled receptors. Neuron 63:27–39. 10.1016/j.neuron.2009.06.014 19607790 PMC2751885

[B3] Alves-Rodrigues A, Leurs R, Willems E, Timmerman H (1996) Binding of clozapine metabolites and analogues to the histamine H3 receptor in rat brain cortex. Arch Pharm (Weinheim) 329:413–416. 8915103 10.1002/ardp.19963290808

[B4] Anderson SA, Michaelides M, Zarnegar P, Ren Y, Fagergren P, Thanos PK, Wang GJ, Bannon M, Neumaier JF, Keller E, Volkow ND, Hurd YL (2013) Impaired periamygdaloid-cortex prodynorphin is characteristic of opiate addiction and depression. J Clin Invest 123:5334–5341. 10.1172/JCI70395 24231353 PMC3859405

[B5] Armbruster BN, Li X, Pausch MH, Herlitze S, Roth BL (2007) Evolving the lock to fit the key to create a family of G protein-coupled receptors potently activated by an inert ligand. Proc Natl Acad Sci U S A 104:5163–5168. 10.1073/pnas.0700293104 17360345 PMC1829280

[B6] Baldessarini RJ, Centorrino F, Flood JG, Volpicelli SA, Huston-Lyons D, Cohen BM (1993) Tissue concentrations of clozapine and its metabolites in the rat. Neuropsychopharmacology 9:117–124. 10.1038/npp.1993.50 8216694

[B7] Boender AJ, de Jong JW, Boekhoudt L, Luijendijk MC, van der Plasse G, Adan RA (2014) Combined use of the canine adenovirus-2 and DREADD-technology to activate specific neural pathways in vivo. PLoS One 9:e95392. 10.1371/journal.pone.0095392 24736748 PMC3988196

[B8] Bull C, Freitas KC, Zou S, Poland RS, Syed WA, Urban DJ, Minter SC, Shelton KL, Hauser KF, Negus SS, Knapp PE, Bowers MS (2014) Rat nucleus accumbens core astrocytes modulate reward and the motivation to self-administer ethanol after abstinence. Neuropsychopharmacology 39:2835–2845. 10.1038/npp.2014.135 24903651 PMC4200494

[B9] Bun H, Disdier B, Aubert C, Catalin J (1999) Interspecies variability and drug interactions of clozapine metabolism by microsomes. Fundam Clin Pharmacol 13:577–581. 10520731 10.1111/j.1472-8206.1999.tb00364.x

[B10] Chang SE, Todd TP, Bucci DJ, Smith KS (2015) Chemogenetic manipulation of ventral pallidal neurons impairs acquisition of sign-tracking in rats. Eur J Neurosci 42:3105–3116. 10.1111/ejn.13103 26469930 PMC4715659

[B11] Chang WH, Lin SK, Lane HY, Wei FC, Hu WH, Lam YW, Jann MW (1998) Reversible metabolism of clozapine and clozapine N-oxide in schizophrenic patients. Prog Neuropsychopharmacol Biol Psychiatry 22:723–739. 9723115 10.1016/s0278-5846(98)00035-9

[B12] Chen X, Choo H, Huang XP, Yang X, Stone O, Roth BL, Jin J (2015) The first structure-activity relationship studies for designer receptors exclusively activated by designer drugs. ACS Chem Neurosci 6:476–484. 10.1021/cn500325v 25587888 PMC4368042

[B13] Conti LH, Costill JE, Flynn S, Tayler JE (2005) Effects of a typical and an atypical antipsychotic on the disruption of prepulse inhibition caused by corticotropin-releasing factor and by rat strain. Behav Neurosci 119:1052–1060. 10.1037/0735-7044.119.4.1052 16187833

[B14] Coward DM (1992) General pharmacology of clozapine. Br J Psychiatry Suppl (17):5–11. 1358127

[B15] Coward P, Wada HG, Falk MS, Chan SD, Meng F, Akil H, Conklin BR (1998) Controlling signaling with a specifically designed Gi-coupled receptor. Proc Natl Acad Sci U S A 95:352–357. 9419379 10.1073/pnas.95.1.352 PMC18222

[B16] Cremers TI, Flik G, Hofland C, Stratford RE Jr. (2012) Microdialysis evaluation of clozapine and N-desmethylclozapine pharmacokinetics in rat brain. Drug Metab Dispos 40:1909–1916. 10.1124/dmd.112.045682 22736307

[B17] Davies MA, Compton-Toth BA, Hufeisen SJ, Meltzer HY, Roth BL (2005) The highly efficacious actions of N-desmethylclozapine at muscarinic receptors are unique and not a common property of either typical or atypical antipsychotic drugs: is M1 agonism a pre-requisite for mimicking clozapine's actions?. Psychopharmacology (Berl) 178:451–460. 10.1007/s00213-004-2017-1 15765260

[B18] Dell’Anno MT, Caiazzo M, Leo D, Dvoretskova E, Medrihan L, Colasante G, Giannelli S, Theka I, Russo G, Mus L, Pezzoli G, Gainetdinov RR, Benfenati F, Taverna S, Dityatev A, Broccoli V (2014) Remote control of induced dopaminergic neurons in parkinsonian rats. J Clin Invest 124:3215–3229. 10.1172/JCI74664 24937431 PMC4071410

[B19] España RA, Oleson EB, Locke JL, Brookshire BR, Roberts DC, Jones SR (2010) The hypocretin-orexin system regulates cocaine self-administration via actions on the mesolimbic dopamine system. Eur J Neurosci 31:336–348. 10.1111/j.1460-9568.2009.07065.x 20039943 PMC2881680

[B20] España RA, Melchior JR, Roberts DC, Jones SR (2011) Hypocretin 1/orexin A in the ventral tegmental area enhances dopamine responses to cocaine and promotes cocaine self-administration. Psychopharmacology 214:415–426. 10.1007/s00213-010-2048-8 20959967 PMC3085140

[B21] Farrell MS, Pei Y, Wan Y, Yadav PN, Daigle TL, Urban DJ, Lee HM, Sciaky N, Simmons A, Nonneman RJ, Huang XP, Hufeisen SJ, Guettier JM, Moy SS, Wess J, Caron MG, Calakos N, Roth BL (2013) A Gαs DREADD mouse for selective modulation of cAMP production in striatopallidal neurons. Neuropsychopharmacology 38:854–862. 10.1038/npp.2012.251 23303063 PMC3671990

[B22] Feifel D, Shilling PD, Melendez G (2011) Clozapine and PD149163 elevate prepulse inhibition in Brown Norway rats. Behav Neurosci 125:268–272. 10.1037/a0022691 21463026 PMC3079344

[B23] Ferguson SM, Eskenazi D, Ishikawa M, Wanat MJ, Phillips PE, Dong Y, Roth BL, Neumaier JF (2011) Transient neuronal inhibition reveals opposing roles of indirect and direct pathways in sensitization. Nat Neurosci 14:22–24. 10.1038/nn.2703 21131952 PMC3058296

[B24] Ferguson SM, Phillips PE, Roth BL, Wess J, Neumaier JF (2013) Direct-pathway striatal neurons regulate the retention of decision-making strategies. J Neurosci 33:11668–11676. 10.1523/JNEUROSCI.4783-12.2013 23843534 PMC3724555

[B25] Gompf HS, Budygin EA, Fuller PM, Bass CE (2015) Targeted genetic manipulations of neuronal subtypes using promoter-specific combinatorial AAVs in wild-type animals. Front Behav Neurosci 9:152 10.3389/fnbeh.2015.00152 26190981 PMC4488755

[B26] Grace PM, Strand KA, Galer EL, Urban DJ, Wang X, Baratta MV, Fabisiak TJ, Anderson ND, Cheng K, Greene LI, Berkelhammer D, Zhang Y, Ellis AL, Yin HH, Campeau S, Rice KC, Roth BL, Maier SF, Watkins LR (2016) Morphine paradoxically prolongs neuropathic pain in rats by amplifying spinal NLRP3 inflammasome activation. Proc Natl Acad Sci U S A 113:E3441–E3450. 10.1073/pnas.1602070113 27247388 PMC4914184

[B27] Guettier JM, Gautam D, Scarselli M, Ruiz de Azua I, Li JH, Rosemond E, Ma X, Gonzalez FJ, Armbruster BN, Lu H, Roth BL, Wess J (2009) A chemical-genetic approach to study G protein regulation of beta cell function in vivo. Proc Natl Acad Sci U S A 106:19197–19202. 10.1073/pnas.0906593106 19858481 PMC2767362

[B28] Huang XF, Tan YY, Huang X, Wang Q (2007) Effect of chronic treatment with clozapine and haloperidol on 5-HT(2A and 2C) receptor mRNA expression in the rat brain. Neurosci Res 59:314–321. 10.1016/j.neures.2007.08.001 17868938

[B29] Jann MW, Grimsley SR, Gray EC, Chang WH (1993) Pharmacokinetics and pharmacodynamics of clozapine. Clin Pharmacokinet 24:161–176. 10.2165/00003088-199324020-00005 8453823

[B30] Jann MW, Lam YW, Chang WH (1994) Rapid formation of clozapine in guinea-pigs and man following clozapine-N-oxide administration. Arch Int Pharmacodyn Ther 328:243–250. 7710309

[B31] Kätzel D, Nicholson E, Schorge S, Walker MC, Kullmann DM (2014) Chemical-genetic attenuation of focal neocortical seizures. Nat Commun 5:3847. 10.1038/ncomms4847 24866701 PMC4050272

[B32] Keith VA, Mansbach RS, Geyer MA (1991) Failure of haloperidol to block the effects of phencyclidine and dizocilpine on prepulse inhibition of startle. Biol Psychiatry 30:557–566. 1834231 10.1016/0006-3223(91)90025-h

[B33] Lin G, McKay G, Hubbard JW, Midha KK (1994) Decomposition of clozapine N-oxide in the qualitative and quantitative analysis of clozapine and its metabolites. J Pharm Sci 83:1412–1417. 7884661 10.1002/jps.2600831010

[B34] Lin G, McKay G, Midha KK (1996) Characterization of metabolites of clozapine N-oxide in the rat by micro-column high performance liquid chromatography/mass spectrometry with electrospray interface. J Pharm Biomed Anal 14:1561–1577. 8877864 10.1016/0731-7085(96)01738-4

[B35] Ma S, Allocca G, Ong-Palsson EK, Singleton CE, Hawkes D, McDougall SJ, Williams SJ, Bathgate RA, Gundlach AL (2016) Nucleus incertus promotes cortical desynchronization and behavioral arousal. Brain Struct Funct. Advance online publication. Retrieved October 13, 2016. doi:10.1007/s00429-016-1230-0. 10.1007/s00429-016-1230-0 27206427

[B36] Marchant NJ, Whitaker LR, Bossert JM, Harvey BK, Hope BT, Kaganoversusky K, Adhikary S, Prisinzano TE, Vardy E, Roth BL, Shaham Y (2016) Behavioral and physiological effects of a novel kappa-opioid receptor-based DREADD in rats. Neuropsychopharmacology 41:402–409. 10.1038/npp.2015.149 26019014 PMC5130116

[B37] Michaelides M, Anderson SA, Ananth M, Smirnov D, Thanos PK, Neumaier JF, Wang GJ, Volkow ND, Hurd YL (2013) Whole-brain circuit dissection in free-moving animals reveals cell-specific mesocorticolimbic networks. J Clin Invest 123:5342–5350. 10.1172/JCI72117 24231358 PMC3859392

[B38] Mizoguchi H, Katahira K, Inutsuka A, Fukumoto K, Nakamura A, Wang T, Nagai T, Sato J, Sawada M, Ohira H, Yamanaka A, Yamada K (2015) Insular neural system controls decision-making in healthy and methamphetamine-treated rats. Proc Natl Acad Sci U S A 112:E3930–E3939. 10.1073/pnas.1418014112 26150496 PMC4517258

[B39] Mosier KE, Song J, McKay G, Hubbard JW, Fang J (2003) Determination of clozapine, and its metabolites, N-desmethylclozapine and clozapine N-oxide in dog plasma using high-performance liquid chromatography. J Chromatogr B Analyt Technol Biomed Life Sci 783:377–382. 12482480 10.1016/s1570-0232(02)00655-4

[B40] Natesan S, Reckless GE, Barlow KB, Nobrega JN, Kapur S (2007) Evaluation of N-desmethylclozapine as a potential antipsychotic–preclinical studies. Neuropsychopharmacology 32:1540–1549. 10.1038/sj.npp.1301279 17164815

[B41] Peters J-U (2012) Polypharmacology in drug discovery. Hoboken, NJ: Wiley.

[B42] Pienaar IS, Gartside SE, Sharma P, De Paola V, Gretenkord S, Withers D, Elson JL, Dexter DT (2015) Pharmacogenetic stimulation of cholinergic pedunculopontine neurons reverses motor deficits in a rat model of Parkinson's disease. Mol Neurodegener 10:47 10.1186/s13024-015-0044-5 26394842 PMC4580350

[B43] Pirmohamed M, Williams D, Madden S, Templeton E, Park BK (1995) Metabolism and bioactivation of clozapine by human liver in vitro. J Pharmacol Exp Ther 272:984–990. 7891353

[B44] Qiu MH, Chen MC, Fuller PM, Lu J (2016) Stimulation of the pontine parabrachial nucleus promotes wakefulness via extra-thalamic forebrain circuit nodes. Curr Biol 26:2301–2312. 27546576 10.1016/j.cub.2016.07.054 PMC5025760

[B45] Ray RS, Corcoran AE, Brust RD, Kim JC, Richerson GB, Nattie E, Dymecki SM (2011) Impaired respiratory and body temperature control upon acute serotonergic neuron inhibition. Science 333:637–642. 10.1126/science.1205295 21798952 PMC3729433

[B46] Redfern CH, Coward P, Degtyarev MY, Lee EK, Kwa AT, Hennighausen L, Bujard H, Fishman GI, Conklin BR (1999) Conditional expression and signaling of a specifically designed Gi-coupled receptor in transgenic mice. Nat Biotechnol 17:165–169. 10.1038/6165 10052353

[B47] Robinson S, Todd TP, Pasternak AR, Luikart BW, Skelton PD, Urban DJ, Bucci DJ (2014) Chemogenetic silencing of neurons in retrosplenial cortex disrupts sensory preconditioning. J Neurosci 34:10982–10988. 10.1523/JNEUROSCI.1349-14.2014 25122898 PMC4131013

[B48] Salmi P, Ahlenius S (1996) Further evidence for clozapine as a dopamine D1 receptor agonist. Eur J Pharmacol 307:27–31. 8831100 10.1016/0014-2999(96)00181-1

[B49] Schotte A, Janssen PF, Megens AA, Leysen JE (1993) Occupancy of central neurotransmitter receptors by risperidone, clozapine and haloperidol, measured ex vivo by quantitative autoradiography. Brain Res 631:191–202. 10.1016/0006-8993(93)91535-Z 7510574

[B50] Scofield MD, Boger HA, Smith RJ, Li H, Haydon PG, Kalivas PW (2015) Gq-DREADD selectively initiates glial glutamate release and inhibits cue-induced cocaine seeking. Biol Psychiatry 78:441–451. 10.1016/j.biopsych.2015.02.016 25861696 PMC4547911

[B51] Sengupta A, Winters B, Bagley EE, McNally GP (2016) Disrupted prediction error links excessive amygdala activation to excessive fear. J Neurosci 36:385–395. 10.1523/JNEUROSCI.3670-15.2016 26758831 PMC6602025

[B52] Sur C, Mallorga PJ, Wittmann M, Jacobson MA, Pascarella D, Williams JB, Brandish PE, Pettibone DJ, Scolnick EM, Conn PJ (2003) N-desmethylclozapine, an allosteric agonist at muscarinic 1 receptor, potentiates N-methyl-D-aspartate receptor activity. Proc Natl Acad Sci U S A 100:13674–13679. 10.1073/pnas.1835612100 14595031 PMC263872

[B53] Swerdlow NR, Bakshi V, Geyer MA (1996) Seroquel restores sensorimotor gating in phencyclidine-treated rats. J Pharmacol Exp Ther 279:1290–1299. 10.1016/0924-977X(96)87794-7 8968353

[B54] Swerdlow NR, Weber M, Qu Y, Light GA, Braff DL (2008) Realistic expectations of prepulse inhibition in translational models for schizophrenia research. Psychopharmacology (Berl) 199:331–388. 10.1007/s00213-008-1072-4 18568339 PMC2771731

[B55] Thomas DR, Dada A, Jones GA, Deisz RA, Gigout S, Langmead CJ, Werry TD, Hendry N, Hagan JJ, Davies CH, Watson JM (2010) N-desmethylclozapine (NDMC) is an antagonist at the human native muscarinic M(1) receptor. Neuropharmacology 58:1206–1214. 20206188 10.1016/j.neuropharm.2010.02.017

[B56] Wang S, Tan Y, Zhang JE, Luo M (2013) Pharmacogenetic activation of midbrain dopaminergic neurons induces hyperactivity. Neurosci Bull 29:517–524. 10.1007/s12264-013-1327-x 23516143 PMC5561950

[B57] Wicker E, Forcelli PA (2016) Chemogenetic silencing of the midline and intralaminar thalamus blocks amygdala-kindled seizures. Exp Neurol 283:404–412. 10.1016/j.expneurol.2016.07.003 27404844 PMC4992629

[B58] Wirtshafter D, Stratford TR (2016) Chemogenetic inhibition of cells in the paramedian midbrain tegmentum increases locomotor activity in rats. Brain Res 1632:98–106. 10.1016/j.brainres.2015.12.014 26707405

[B59] Witten IB, Steinberg EE, Lee SY, Davidson TJ, Zalocusky KA, Brodsky M, Yizhar O, Cho SL, Gong S, Ramakrishnan C, Stuber GD, Tye KM, Janak PH, Deisseroth K (2011) Recombinase-driver rat lines: tools, techniques, and optogenetic application to dopamine-mediated reinforcement. Neuron 72:721–733. 10.1016/j.neuron.2011.10.028 22153370 PMC3282061

[B60] Wong G, Kuoppamäki M, Hietala J, Lüddens H, Syvälahti E, Korpi ER (1996) Effects of clozapine metabolites and chronic clozapine treatment on rat brain GABAA receptors. Eur J Pharmacol 314:319–323. 8957253 10.1016/s0014-2999(96)00671-1

[B61] Yau JO, McNally GP (2015) Pharmacogenetic excitation of dorsomedial prefrontal cortex restores fear prediction error. J Neurosci 35:74–83. 10.1523/JNEUROSCI.3777-14.2015 25568104 PMC6605253

[B62] Yorgason JT, España RA, Jones SR (2011) Demon voltammetry and analysis software: analysis of cocaine-induced alterations in dopamine signaling using multiple kinetic measures. J Neurosci Methods 202:158–164. 10.1016/j.jneumeth.2011.03.001 21392532 PMC3149733

[B63] Zhu H, Pleil KE, Urban DJ, Moy SS, Kash TL, Roth BL (2014) Chemogenetic inactivation of ventral hippocampal glutamatergic neurons disrupts consolidation of contextual fear memory. Neuropsychopharmacology 39:1880–1892. 10.1038/npp.2014.35 24525710 PMC4059896

